# Correlates of Immunity to Filovirus Infection

**DOI:** 10.3390/v3070982

**Published:** 2011-06-27

**Authors:** Steven B. Bradfute, Sina Bavari

**Affiliations:** United States Army Medical Research Institute of Infectious Diseases, 1425 Porter Street, Fort Detrick, Maryland, MD 21702, USA; E-Mail: steven.bradfute@us.army.mil

**Keywords:** filovirus, ebola, marburg, immunity

## Abstract

Filoviruses can cause severe, often fatal hemorrhagic fever in humans. Recent advances in vaccine and therapeutic drug development have provided encouraging data concerning treatment of these infections. However, relatively little is known about immune responses in fatal *versus* non-fatal filovirus infection. This review summarizes the published literature on correlates of immunity to filovirus infection, and highlights deficiencies in our knowledge on this topic. It is likely that there are several types of successful immune responses, depending on the type of filovirus, and the presence and timing of vaccination or drug treatment.

## 1. Introduction

Filoviruses are enveloped, single-stranded, negative-sense RNA viruses that can cause lethal hemorrhagic fever [1]. Three distinct groups of filoviruses have been described: Ebolaviruses, marburgviruses, and the newly discovered cuevaviruses. There are five currently known ebolaviruses—Ebola (EBOV, previously called Zaire), Sudan (SUDV), Bundibugyo (BDBV), Taï Forest (TAFV, previously called Côte d’Ivoire), and Reston (RESTV); two marburgviruses—Marburg (MARV) and Ravn (RAVV); and one cuevavirus, Lloviu (LLOV) [2]. EBOV and marburgviruses appear to have similar lethality in humans, with rates of approximately 70–90%. SUDV lethality in humans is ≈50%, and BDBV is ≈30%. No human deaths have been attributed to TAFV, RESTV, or LLOV. Filoviruses are endemic in Central Africa and the Philippines, and are NIAID Category A Priority Pathogens due to their lethality, potential for misuse, and ability to be aerosolized. Bats are possible reservoirs for filoviruses, and are thought to spread disease to humans either directly or through infection of nonhuman primates (NHPs) and possibly swine [1,3–7].

A number of vaccines have successfully protected NHPs from filovirus infection (reviewed in [8]). Additionally, anti-sense and RNA interference therapeutics against EBOV or MARV can be protective against NHP infection [9,10]. However, no treatment or vaccine has been proven to be effective in human filovirus infection.

Wild-type filoviruses are lethal in untreated NHPs, making comparison of successful *versus* unsuccessful immune responses difficult, due to a lack of a significant number of survivors. Wild-type filoviruses are generally not lethal in mice and guinea pigs, but there are lethal mouse-adapted (EBOV, RAVV) and guinea pig-adapted (EBOV, MARV, RAVV) models available [11–15]. Mice infected via the subcutaneous (s.c.) route with mouse-adapted EBOV (ma-EBOV) survive infection, whereas intraperitoneal (i.p.) infection is lethal [11]. Although it is not known why route of infection alters survival outcome, protection is correlated with differential cytokine expression, as described in the next section. Additionally, mice with decreased levels of CD45 expression (CD45^lo^) are resistant to maEBOV infection, and this protection correlates with altered cytokine expression and a requirement for CD8+ T cells and IFN-gamma (see below) [16]. The use of lethal and non-lethal filovirus mouse models allow for analysis of protective immune responses without therapeutic treatment or vaccination. Correlates of immunity to filovirus infection have also been studied in vaccination experiments in NHPs, mice, and guinea pigs. These studies have yielded results that suggest there are several ways the immune system can protect against filoviruses infection.

This review aims to collate the available published data and summarize what is known about immune responses to filovirus infection, and to highlight areas for future research to close the gaps in knowledge on this topic.

## 2. Discussion

### 2.1. Cytokines

Type I interferon (IFN) is essential in controlling filovirus infection. Adult, immunocompetent wild-type mice are not susceptible to wild-type filovirus infection; however, inhibition of type I IFN (via knockout of the IFN alpha/beta receptor I, STAT1, or antibody-mediated depletion of IFN alpha and IFN beta) results in lethal infection with most wild-type filoviruses [17]. Mouse-adapted EBOV likely acquired its lethality in mice by mutations that abrogated mouse type I IFN responses [18]. Additionally, induction of type I IFN protects mice from otherwise lethal maEBOV infection [19,20]. Treatment of NHPs with IFN-alpha2 prolongs time-to-death in EBOV- or MARV-infected NHP [21,22]. Although type I IFN is sometimes elevated in lethal infection (see below), it is often detected later in infection, perhaps too late to be effective. On a molecular level, certain filoviral genes (VP35, VP24, and VP40) inhibit type I IFN function through a variety of mechanisms [23–38]. This topic is more thoroughly reviewed by [39].

Due to the sporadic nature of filovirus outbreaks, and the remote locations where the viruses are endemic, it is difficult to obtain samples from infected humans. Nonetheless, a few studies of cytokine expression in human fatal and non-fatal EBOV and SUDV infection have been published. It is very difficult to directly compare cytokine responses between survivors and non-survivors in human infections. Pre-existing endemic infections (such as HIV or parasites) could impact survival after filovirus infection, but these variables are often not analyzed. Most published studies have compared samples from these groups based on time of symptom onset. Although this is a reasonable comparison, it does not account for the possibility that survivors or non-survivors may have differences in immune responses prior to symptom onset. For example, survivors may have more robust type I IFN responses before symptom onset compared to non-survivors. Sampling of cytokine expression based on onset of symptoms would then fail to detect early responses that may control the overall outcome of infection. Therefore, time of infection is a more accurate basis to compare immune responses between survivors and non-survivors. Of course, it is nearly impossible to determine time of infection in human outbreak settings, highlighting one advantage of using animal models to analyze immune responses. The human cytokine data are vital and informative, but must be analyzed with these limitations in mind.

Although some of the human data are contradictory, the limited data suggest that fatal EBOV infection is correlated with an increase in pro-inflammatory cytokines (such as tumor necrosis factor (TNF)-alpha, IFN-gamma, IL-6, IL-8, IL-1 beta, MIP-1 alpha, MIP-1 beta, MCP1, *etc.*), whereas non-fatal infection lacks this explosion of cytokine release [40–43]. IL-10 production is also elevated during lethal EBOV infection, possibly due to attempts to dampen the rampant inflammatory response [44]. Interestingly, early and late IL-10 production is decreased in maEBOV-resistant CD45^lo^ mice compared to wild-type mice, suggesting that temporal regulation of IL-10 may be important in controlling filovirus infection [16]. NHP studies of lethal EBOV [45–47] and RESTV [48] infection confirm the increase in pro-inflammatory cytokine production. Possible asymptomatic EBOV infection in humans is correlated with strong but transient early pro-inflammatory cytokine production [49,50], again suggesting that temporal control of cytokine expression is important in controlling EBOV infection. Increased type I IFN and TNF-alpha concentrations also correlated with fatal MARV infection in NHPs [51], and neutralization of TNF-alpha with antibody improved survival of guinea pigs after MARV infection [52]. Notably, TNF-alpha and IFN-gamma are not elevated in fatal human SUDV infections as they are in fatal human EBOV infections, suggesting possible differences in immune responses to different filovirus species [53,54].

Filovirus mouse models are useful for immune correlate studies, as both lethal and non-lethal models exist. Mice infected with lethal (mouse-adapted) RAVV generated higher levels of pro-inflammatory cytokines and chemokines (such as IFN-gamma, IL-5, IL-12, MCP-1, MIG, and IFN-alpha) than mice infected with non-lethal (wild-type) RAVV [15]. Mice lethally infected with maEBOV (i.p. infection) generated higher TNF-alpha and MCP-1 levels, but lower IFN-gamma and IFN-alpha, compared to non-lethal (s.c.) infection [55]. Mice genetically resistant to maEBOV infection (CD45^lo^ mice), require IFN-gamma for protection [16]. Similarly, IFN-gamma is required for successful virus-like particle (VLP) vaccination against maEBOV in wild-type mice [56].

Together, these data suggest that *temporal* regulation of cytokine production is important in controlling filovirus infection. One model proposes that inhibition of early type I IFN responses, but an increase in other pro-inflammatory cytokine/chemokine expression, results in fatal filoviral infection [23]. In this model, belated type I IFN responses would result in delayed adaptive immune responses and decreased antiviral innate responses; the later deluge of pro-inflammatory cytokines, such as TNF-alpha, would contribute to organ damage and vascular leakage in lethal infection. However, a tightly controlled, transient early type I IFN and pro-inflammatory cytokine response would induce protective antiviral innate and adaptive immune responses. It is important to note that there are some contradictions in cytokine levels reported between SUDV and EBOV, suggesting that direct comparison across different filoviruses may not be possible [40–43,53,54]. It is also notable that induction of massive pro-inflammatory cytokine expression occurs in human infections, as well as NHP and mouse models of filovirus infections, revealing the usefulness of animal models to study human disease.

### 2.2. Lymphocyte Apoptosis

Widespread and profound lymphocyte apoptosis is found in fatal, but not non-fatal, filovirus infection. However, lymphocytes are not productively infected with filoviruses. Lymphocyte death was first discovered by microscopy in the organs of human patients that succumbed to EBOV infection [57,58]. Apoptosis in peripheral blood cells, as measured by DNA cleavage in PBMC, was found in fatal, but non non-fatal, EBOV infection in humans [40]. NHPs or mice lethally infected with EBOV or MARV also display lymphocyte apoptosis (as measured by conventional microscopy, electron microscopy, or TUNEL staining) in multiple tissues [15,45,59–61]. EBOV lymphocyte apoptosis has been found to correlate with increased Fas/FasL and/or TRAIL expression [42,45,46,62]. Additionally, transgenic mice that survive EBOV infection do not have profound lymphocyte apoptosis, whereas those that succumb to infection do [16].

Findings such as these led to a hypothesis that filovirus-induced bystander lymphocyte apoptosis results in the elimination of adaptive immune responses and subsequent overwhelming viral pathogenesis [40,45,61,63]. However, ensuing experiments demonstrated that lethally infected mice generated a maEBOV-specific CD8+ T cell response despite the presence of T cell apoptosis [60]. This response correlated with the appearance of lymphoblasts, rebound of peripheral blood CD8+ T cell numbers, and expression of activation markers (increased CD44 and decreased CD62L and CD127). Notably, adoptive transfer of purified CD8+ T cells from lethally infected mice protected naïve recipients from subsequent maEBOV infection [60]. This occurred despite concurrent lymphocyte apoptosis in lymphoid organs. The appearance of lymphoblasts, increased lymphocyte CD44 expression, and late-stage increase in peripheral lymphocyte counts have been reported in human and/or NHP EBOV studies [45,54,62,64,65]. Additionally, specific antibody responses can be found in many EBOV and SUDV lethally-infected humans [40,66–68] (Table 1). These data suggest that a functional and specific CD8+ T cell (and perhaps B cell) response can be generated in lethal infection, despite massive bystander lymphocyte apoptosis.

Transgenic and knockout mice were used to directly test whether lymphocyte apoptosis is important for lethal filovirus infection. maEBOV-induced lymphocyte apoptosis was shown to occur via both the extrinsic (death receptor) and intrinsic apoptotic pathways in lethally infected mice [69]. However, the elimination of maEBOV-induced lymphocyte apoptosis (in bcl-2 transgenic mice) did not protect animals from lethal infection, suggesting that lymphocyte apoptosis is not a major factor in EBOV pathogenesis [69]. In fact, bcl-2 transgenic mice had higher viral titers and decreased CD8+ T-cell responses compared to wild-type mice.

Together, these data suggest that EBOV-induced lymphocyte apoptosis does not abrogate adaptive immune responses, and is not required for pathogenesis. Why does it occur? It is possible that the high levels of pro-inflammatory cytokine expression late in lethal filovirus infection leads to bystander lymphocyte apoptosis, and is a byproduct of fatal infection but not a cause. Experiments conducted in an acute, non-lethal lymphocytic choriomeningitis (LCMV) infection mouse model have shown that both LCMV specific and non-specific CD8+ T cells undergo apoptosis during infection, despite a successful CD8+ T-cell response [70,71]. Interestingly, there is evidence that this apoptosis is mediated by type I IFN production [71]. Future experiments should focus on whether the apoptotic T and B cells in filovirus infection are filovirus-specific.

### 2.3. B Cells and Antibody

Antibody levels are generally thought to be low or absent in fatal filoviral infection, leading to the hypothesis that suppression of B cell responses and antibody production correlate with fatality. However, a review of the limited available literature (Table 1) shows that antibody responses are found in many fatal EBOV and SUDV human infections, at levels similar to those of some survivors [40,66–68]. These data suggest that antibody responses can be generated in lethal infection, and the mere presence or absence of antibody does not necessarily correlate with lethality. However, the data do not rule out the possibility that survivors generate more *functional* antibody than non-survivors.

Successful vaccination against filovirus infection in NHPs generates a range of antibody titers, depending on the vaccine platform [72–79]; most platforms do not generate substantial *in vitro* neutralizing antibody titers. Protection against EBOV challenge using adenovirus-based vaccines correlates with total antibody titers; all NHPs that generated a certain level of antibody titer were protected from challenge, whereas those having lower titers were only partially protected [80]. On the other hand, vaccination of NHPs with replication-competent vesicular stomatitis Indiana virus (VSV)-based filovirus vaccines generated low levels of antibody, but vaccination was protective when given pre- or post- infection against a number of filoviruses [81–86]. Virus-like particle (VLP) vaccines generated moderate to high levels of antibody in NHPs, along with moderate levels of neutralizing antibody, against MARV and EBOV [72,73]. B cells were required for protection against maEBOV in VLP-vaccinated mice, but this has not been tested in any other filovirus vaccine platform [87]. In an extreme example, vaccination against EBOV and SUDV protected NHPs against BDBV infection, even though no antibodies against BDBV were detected [79]. While these findings must be confirmed, it appears that the antibody level required to protect against filovirus infection probably differs according to the vaccination platform and regimen, as well as the particular filovirus being tested.

Transfer of IgG+ convalescent whole blood to EBOV-infected human patients protected 8/9 from lethal infection (compared to 20% survival in untreated patients) [88]. Although this study was not controlled, and the transfused patients received better care than the untreated patients, it did suggest the possibility that antibody could protect against filovirus infection. Passive transfer of whole sera or monoclonal antibodies have been shown to protect mice or guinea pigs from lethal EBOV or MARV infection [13,21,89–98]. Interestingly, passive transfer experiments showed that *in vitro* neutralizing antibody activity was not necessarily required for antibody-mediated protection of maEBOV infection in mice or MARV infection in guinea pigs [89,92,96]. Passive transfer of immune horse sera protected baboons from EBOV infection [97,99,100]; however, other studies in EBOV-infected macaques did not show protection with passive transfer of a monoclonal antibody, immune IgG, or convalescent whole blood [21,101,102]. These mixed results implore the initiation of additional studies to rescue NHP from filovirus infection by passive transfer of antibodies or immune sera.

There is virtually no information on how antibody might aid in protection against filovirus infection. As mentioned above, most successful vaccinations in NHP filovirus models do not generate *in vitro* neutralizing antibodies, and neutralization itself is not required for protection in passive transfer experiments. There is little to no information on complement fixation, ADCC, or opsonization capabilities of antibodies generated in vaccinated or surviving animals or humans in response to filovirus infections. Measurement of antibody titers against filovirus antigen does not measure how *functional* the antibody is; many pathogens (such as HIV-1) induce antibody production against non-protective epitopes. Therefore, we really have no idea *how* antibody might protect against filovirus infection. More thorough investigations on functionality of antibody would likely address the varied results obtained from the vaccination studies listed above. It is likely that antibodies induced in different ways will act through different mechanisms to induce protection; therefore, it is important to note that failure of a particular antibody preparation to protect against filovirus infection does not necessarily mean that antibody is not a potential therapeutic regimen. Additionally, further work to establish the importance and requirement for antibodies in protection against different filoviruses is warranted.

### 2.4. T Cells

Humans that survive SUDV infection have been shown to generate higher percentages of phenotypically activated (HLA-DR+) CD8+ T cells than non-survivors [54]. Similarly, CD45^lo^ mice are resistant to maEBOV-induced lethality, and generate higher percentages of phenotypically activated (CD44 high) CD8+, but not CD4+, T cells [16]. Depletion of CD8a+ T cells in these mice leads to lethal maEBOV infection.

CD8+ T cells and CD4+ T cells are required for VLP vaccine-mediated protection against maEBOV in mice [87]. However, depletion of CD8+ T cells during VSV vaccination (but not challenge) against maEBOV does not abrogate protection in mice [91]. CD8+ T cells are required for protection in mice infected s.c. with maEBOV, and this protection requires perforin, but not Fas or IFN-gamma [103,104]. Vaccination of mice with Venezuelan equine encephalitis virus replicons (VRP)-based vectors generated multiple epitope-specific CD8+ T cells that were protective after expansion and subsequent adoptive transfer [105,106]. Similarly, mice infected with wild-type, non-lethal RAVV generated epitope-specific CD8+ T cells; adoptive transfer of splenocytes from these mice, after expansion *in vitro* with peptide epitopes, protected recipient mice from subsequent mouse-adapted RAVV infection [106].

In NHP studies, different protective vaccination platforms generate various levels of CD4+ and/or CD8+ T-cell responses, ranging from non-existent to profound [73,74,76–79,107,108]. Vaccination with EBOV VLPs generated robust T-cell cytokine responses [73]. A DNA prime/adenovirus-GP_1,2_ boost (DNA/AdV) vaccine regimen that is protective against EBOV infection in NHPs generated memory CD4+, but not CD8+, T cells that proliferated *in vitro* after stimulation with antigen [78]. CD8+ T-cell IFN-gamma responses were, however, present after DNA/AdV vaccination [77]. Subsequent studies with AdV only-vaccinated NHP did not demonstrate a clear correlation with antigen-specific memory CD8+ or CD4+ T-cell cytokine expression and protection against EBOV infection [107]. Similarly, successful vaccination against MARV generated multiple cytokine-producing CD4+ and CD8+ T cells, but there was no clear correlation with the magnitude of memory T-cell generation and protection against clinical signs after challenge [108]. Vaccination against EBOV and SUDV did not generate detectable antibodies against BDBV, but did induce CD4+ and CD8+ T-cell responses against BDBV [79]. This was sufficient for protection against BDBV challenge, suggesting generation of memory T cells may possibly be sufficient for protection [79]. Vaccination with a VSV-based vector expressing EBOV or MARV GP_1,2_ was protective in NHPs, but did not generate specific CD4+ or CD8+ T-cell responses [74]. Similarly, human parainfluenza virus type 3-based EBOV vaccines induced low to undetectable T-cell responses, yet still protect against infection [76].

Therefore, the available data suggest that that requirement of T cell subsets for effective protection against filovirus infection may differ depending on the vaccine platforms used to induce protection. Protection of unvaccinated individuals from filovirus infection may also heavily depend on both CD4+ and CD8+ T cells’ responses, although there is insufficient data to conclusively prove this hypothesis.

### 2.5. NK Cells

NK cells have been hypothesized to be important in controlling filovirus infection, since loss of peripheral NK cell numbers is found in lethally EBOV-infected NHPs [62], and NK cells are necessary for VLP vaccination against maEBOV infection in mice [109]. Furthermore, human NK cells activated with EBOV VLPs *in vitro* can kill EBOV- or MARV-infected DCs, and this cytotoxicity may occur via perforin or FasL and is partially reliant on the activating receptor NKp30 [110]. On the other hand, humans that survive EBOV infection were more likely to carry an “activating” repertoire of the killer immunoglobulin-like receptor genes KIR2DS3 and KIR2DS1 when compared to those that succumb to infection [111]. CD45^lo^ mice resistant to EBOV infection, however, do not require NK cells for protection [16]. NK cell activation is complex, and hinges on a complex interplay of activating and inhibitory receptors. Therefore, it is still uncertain what role NK cells play in protection against filovirus infection.

### 2.6. HLA

One study analyzed human leukocyte antigen-B (HLA-B) alleles in 77 humans that either succumbed to or survived SUDV infection. Survival correlated with HLA-B*07 (11/11 infected individuals carrying this allele survived) and HLA-B-*14 (4/4 survivors), while HLA-B*67 (0/8 survivors) and HLA-B*15 (4/17 survivors) alleles were associated with lethality [112]. This study suggests that the repertoire of antigen presentation on MHC I is an important factor in combating SUDV infection, further highlighting the possible importance of CD8+ T cells in filovirus infection.

### 2.7. Macrophages and Dendritic Cells

Alveolar macrophages infected *in vitro* with EBOV have decreased MHC I levels and increased IL-1beta, IL-6, IL-8, MIP-1 alpha, and TNF alpha production relative to mock-infected cells [113]. Similarly, monocytes infected *in vitro* with EBOV, MARV, or RESTV express increased TNF, IL-6, IL-8, and gro-alpha mRNA relative to mock-infected cells [114]. Macrophages and/or monocytes have been shown to be infected *in vivo* with TAFV, EBOV, MARV, and RESTV [12,64,115–119]. This conclusion was reached by the detection of inclusion bodies in and virus budding from these cells; the mere detection of viral RNA or antigen is not sufficient for determination of viral replication in phagocytic cells since they can engulf previously infected cells and debris.

Filoviruses can replicate *in vitro* in human myeloid dendritic cells (DCs). EBOV and MARV infection of human conventional DCs (cDC) led to viral replication, but not secretion of IL-6, RANTES, IL-10, IL-1b, IL-8, IL-12, or IFN-alpha [120]. Infection also inhibited expression of IFN-alpha in response to subsequent VRP infection in a filoviral replication-independent manner. Inactivated MARV or EBOV treatment of DCs decreased allogeneic T-cell proliferation relative to mock-treated DC [120]. EBOV-infected DCs had decreased CD86, CD83, and HLA-DR expression compared to lipopolysaccharide (LPS)-treated cells, but still had increased levels of CD80 and CD86 compared to mock-infected cells. A separate study also found EBOV replication in human myeloid DCs *in vitro*, and did not find increased levels of TNF-alpha, IL-6, IL-1b, IL-10, MIP-1 beta, although slight increases were found in IL-8, MIP-1a and MCP-1 relative to mock-infected cells [121]. CD40, CD80, and CD86 levels were similar between EBOV-infected and mock-infected cells. EBOV-infected DCs were inhibited in their ability to induced allogeneic T-cell proliferation relative to LPS-treated DCs, but were still increased compared to mock-infected cells. In this study, however, inactivated EBOV-treated DCs were similar to mock-infected DCs in their ability to induce allogeneic T-cell proliferation.

It is important to note that the *in vitro* DC experiments described above were conducted with conventional DCs. A recent study suggested that plasmacytoid DC (pDC), which are major producers of type I IFN, are resistant to EBOV infection *in vitro* [122]. These data highlight the importance of studying different DC populations in attempting to explain the roles of these cells in filovirus infection.

The data for filoviral infection of DC *in vivo* is scant. One study described detection of EBOV antigen or RNA in DCs (as identified by morphology and DC-SIGN staining) in tissue sections from infected NHP [45]. There has been no analysis of DC subtypes or the frequency of DC infection *in vivo.*

Together, the data suggest that infectious EBOV or MARV do not increase expression of co-stimulatory markers or many pro-inflammatory cytokines in cDC, whereas infected macrophages do produce pro-inflammatory cytokines, at least *in vitro*. Infection of cDC with EBOV increased their ability to induce T-cell proliferation, but not to the level of LPS-stimulated cDC. However, there are not sufficient data to predict the overall effect of filovirus infection on cDC function. It does appear that infection of macrophages *versus* cDC may induce different cytokine responses in these cells [123].

### 2.8. Control of Viral Replication

Most reports have shown that viremia is lower in non-fatal filoviral infection compared to fatal infection. This has been shown in humans that survive SUDV infection [54], mice or guinea pigs surviving EBOV infection after treatment with antivirals that inhibit replication [124,125], and guinea pigs or mice infected with lethal or non-lethal EBOV [11,12]. Similarly, mice infected with maRAVV had higher viral titers in sera and tissues compared to mice infected with wild-type RAVV [15]. A study showing decreased lethality in EBOV or MARV-infected NHP after administration of antisense against viral genes found that survival roughly correlated with decreased viral titer; however, some animals that survived had similar peak viremias to some animals that succumbed [9]. Similarly, resistant CD45^lo^ mice have similar viral titers through day 7 of maEBOV infection when compared to susceptible wild-type mice [16], and some antibody-treated guinea pigs that survive EBOV infection have similar viremias to some animals that succumb to infection [93]. These data indicate that viral replication by itself is not always a cause of pathogenesis; manipulation of cells to respond properly to viral infection may be as important as early control of viral replication.

### 2.9. Gaps

There are significant gaps in our knowledge of immune responses to filovirus infection. There are no data on immune responses to marburgvirus infection in humans. Genetic analysis of PMBC from humans that survived SUDV or EBOV infection *versus* those that succumbed has revealed possible links between HLA and KIR haplotypes and survival [111,112]. Given the advanced state of genome sequencing technology, broader analysis of genetic factors should be performed to advance the knowledge of correlates of immunity against filovirus infection.

Development of immunological tools in filovirus infection is required to be able to answer more complex questions of filovirus immunity. The use of tetramers to detect antigen-specific T cells would greatly aid our understanding of the ability of different vaccines to induce T-cell responses. The creation of transgenic mice with filovirus-specific T or B cells would allow for more elegant studies of immune responses to filovirus infection.

Transgenic and knockout mouse models are invaluable in testing correlates of immunity in filovirus vaccine platforms, as has been demonstrated in VLP vaccination [87]. The available data suggest that there are many different ways for the immune system to successfully combat filovirus infection. For example, different vaccine platforms generate different types of antibody and cell-mediated immune responses [8]. Other filoviral vaccine platforms should be tested in knockout and transgenic mice to discover which immune pathways are required for protection. Furthermore, antibody-mediated depletion of immune cells and cytokines in vaccinated or drug-treated NHPs would shed light on the importance of immune components in filovirus infection in this model.

There is no conclusive evidence that DCs are major targets for filoviral replication *in vivo.* Neither is there any data analyzing *function* of DCs or macrophages *in vivo* after infection. Although *in vitro* studies are important, *in vivo* studies are imperative for confirming how filoviruses affect macrophage and DC populations.

As discussed above, there is conflicting data over whether or not passive transfer of convalescent sera or antibody can be protective in NHP or human filoviral infections. This is possibly due to the varied effectiveness of individual antibody preparations, the animal models used, the virus species, or a combination of these. Additional experiments in NHPs are crucial for exploring this topic. Further studies on how antibody might protect (through neutralization, complement fixation, opsonization, ADCC, *etc.*) from filovirus infection would advance understanding of the feasibility of passive transfer. The possibility of using passive transfer to protect against filovirus infection is enticing, as it would provide a simple, well-characterized therapeutic platform for treatment.

The use of mice resistant to EBOV infection (CD45^lo^ mice, mice infected s.c., or mice infected with wild-type EBOV) or RAVV infection (wild-type RAVV *versus* mouse-adapted RAVV) provide attractive models to compare successful *versus* unsuccessful immune responses to infection. Initial studies using these models have yielded information on immune responses in lethal *versus* non-lethal infections [11,15]. Further studies using these models would be helpful in generating information on immune response to filovirus infection.

## 3. Conclusions

The components required for successful immune responses to filovirus infection are likely to vary, depending on the virus strain and type of vaccination or therapeutic treatment. This is highlighted by the varied antibody and T cell responses generated in different protective vaccination platforms. Although there are currently not enough data for definitive conclusions, it appears that early, transient, and tightly regulated pro-inflammatory and type I IFN cytokine expression correlates with control of viral pathogenesis. MHC I and NK cell receptor genotyping suggests that genetic factors may also play a role in protection against filovirus infection. *In vivo* data on macrophage and DC infection and responses to filovirus infection are lacking, as are comparative studies on immune responses to different filoviruses. The use of lethal and non-lethal animal models is invaluable to fill the gaps in our understanding of correlates of immunity to filovirus infection.

## Figures and Tables

**Table 1:** Anti-ebolavirus antibodies in fatal human cases. The literature was surveyed for reports of anti-filovirus antibodies in the serum of lethally-infected human patients. Four studies were found, and are reported here. In each case, the lowest dilution of serum tested was 1:100. NR, not reported.

**Ebolavirus**	**IgM+**	**IgG+**	**Reference**
SUDV	2/3 (67%)	2/3 (67%)	[67]
SUDV	NR	6/27 (22%)	[68]
EBOV	3/7 (43%)	4/7 (57%)	[66]
EBOV	1/3 (33%)	0/3 (0%)	[40]
**Total SUDV**	2/3 (67%)	8/30 (27%)	
**Total EBOV**	4/10 (40%)	4/10 (40%)	
**Total Combined**	6/13 (46%)	12/40 (30%)	

## References

[1] Kuhn JH (2008). Filoviruses. A compendium of 40 years of epidemiological, clinical, and laboratory studies. Arch. Virol. Suppl..

[2] Kuhn JH, Becker S, Ebihara H, Geisbert TW, Johnson KM, Kawaoka Y, Lipkin WI, Negredo AI, Netesov SV, Nichol ST (2010). Proposal for a revised taxonomy of the family Filoviridae: Classification, names of taxa and viruses, and virus abbreviations. Arch. Virol..

[3] Barrette RW, Metwally SA, Rowland JM, Xu L, Zaki SR, Nichol ST, Rollin PE, Towner JS, Shieh WJ, Batten B (2009). Discovery of swine as a host for the Reston ebolavirus. Science.

[4] Leroy EM, Epelboin A, Mondonge V, Pourrut X, Gonzalez JP, Muyembe-Tamfum JJ, Formenty P (2009). Human Ebola outbreak resulting from direct exposure to fruit bats in Luebo, Democratic Republic of Congo, 2007. Vector Borne Zoonotic Dis..

[5] Pourrut X, Delicat A, Rollin PE, Ksiazek TG, Gonzalez JP, Leroy EM (2007). Spatial and temporal patterns of Zaire ebolavirus antibody prevalence in the possible reservoir bat species. J. Infect. Dis..

[6] Pourrut X, Souris M, Towner JS, Rollin PE, Nichol ST, Gonzalez JP, Leroy E (2009). Large serological survey showing cocirculation of Ebola and Marburg viruses in Gabonese bat populations, and a high seroprevalence of both viruses in Rousettus aegyptiacus. BMC Infect. Dis..

[7] Towner JS, Pourrut X, Albarino CG, Nkogue CN, Bird BH, Grard G, Ksiazek TG, Gonzalez JP, Nichol ST, Leroy EM (2007). Marburg virus infection detected in a common African bat. PLoS ONE.

[8] Bradfute SB, Dye JM, Bavari S (2011). Filovirius vaccines. Hum. Vaccin..

[9] Warren TK, Warfield KL, Wells J, Swenson DL, Donner KS, Van Tongeren SA, Garza NL, Dong L, Mourich DV, Crumley S (2010). Advanced antisense therapies for postexposure protection against lethal filovirus infections. Nat. Med..

[10] Geisbert TW, Lee AC, Robbins M, Geisbert JB, Honko AN, Sood V, Johnson JC, de Jong S, Tavakoli I, Judge A (2010). Postexposure protection of non-human primates against a lethal Ebola virus challenge with RNA interference: A proof-of-concept study. Lancet.

[11] Bray M, Davis K, Geisbert T, Schmaljohn C, Huggins J (1998). A mouse model for evaluation of prophylaxis and therapy of Ebola hemorrhagic fever. J. Infect. Dis..

[12] Connolly BM, Steele KE, Davis KJ, Geisbert TW, Kell WM, Jaax NK, Jahrling PB (1999). Pathogenesis of experimental Ebola virus infection in guinea pigs. J. Infect. Dis..

[13] Hevey M, Negley D, Geisbert J, Jahrling P, Schmaljohn A (1997). Antigenicity and vaccine potential of Marburg virus glycoprotein expressed by baculovirus recombinants. Virology.

[14] Siegert R, Shu HL, Slenczka HL, Peters D, Muller G (1968). The aetiology of an unknown human infection transmitted by monkeys (preliminary communication). Ger. Med. Mon..

[15] Warfield KL, Bradfute SB, Wells J, Lofts L, Cooper MT, Alves DA, Reed DK, VanTongeren SA, Mech CA, Bavari S (2009). Development and characterization of a mouse model for Marburg hemorrhagic fever. J. Virol..

[16] Panchal RG, Bradfute SB, Peyser BD, Warfield KL, Ruthel G, Lane D, Kenny TA, Anderson AO, Raschke WC, Bavari S (2009). Reduced levels of protein tyrosine phosphatase CD45 protect mice from the lethal effects of Ebola virus infection. Cell Host Microbe.

[17] Bray M (2001). The role of the Type I interferon response in the resistance of mice to filovirus infection. J. Gen. Virol..

[18] Ebihara H, Takada A, Kobasa D, Jones S, Neumann G, Theriault S, Bray M, Feldmann H, Kawaoka Y (2006). Molecular determinants of Ebola virus virulence in mice. PLoS Pathog..

[19] Bray M, Driscoll J, Huggins JW (2000). Treatment of lethal Ebola virus infection in mice with a single dose of an *S*-adenosyl-l-homocysteine hydrolase inhibitor. Antivir. Res..

[20] Bray M, Raymond JL, Geisbert T, Baker RO (2002). 3-deazaneplanocin A induces massively increased interferon-alpha production in Ebola virus-infected mice. Antivir. Res..

[21] Jahrling PB, Geisbert TW, Geisbert JB, Swearengen JR, Bray M, Jaax NK, Huggins JW, LeDuc JW, Peters CJ (1999). Evaluation of immune globulin and recombinant interferon-alpha2b for treatment of experimental Ebola virus infections. J. Infect. Dis..

[22] Kolokol'tsov AA, Davidovich IA, Strel'tsova MA, Nesterov AE, Agafonova OA, Agafonov AP (2001). The use of interferon for emergency prophylaxis of marburg hemorrhagic fever in monkeys. Bull. Exp. Biol. Med..

[23] Chang TH, Kubota T, Matsuoka M, Jones S, Bradfute SB, Bray M, Ozato K (2009). Ebola Zaire virus blocks type I interferon production by exploiting the host SUMO modification machinery. PLoS Pathog..

[24] Talon J, Horvath CM, Polley R, Basler CF, Muster T, Palese P, Garcia-Sastre A (2000). Activation of interferon regulatory factor 3 is inhibited by the influenza A virus NS1 protein. J. Virol..

[25] Prins KC, Delpeut S, Leung DW, Reynard O, Volchkova VA, Reid SP, Ramanan P, Cardenas WB, Amarasinghe GK, Volchkov VE Mutations abrogating VP35 interaction with double-stranded RNA render Ebola virus avirulent in guinea pigs. J. Virol..

[26] Reid SP, Cardenas WB, Basler CF (2005). Homo-oligomerization facilitates the interferon-antagonist activity of the ebolavirus VP35 protein. Virology.

[27] Mateo M, Reid SP, Leung LW, Basler CF, Volchkov VE (2010). Ebolavirus VP24 binding to karyopherins is required for inhibition of interferon signaling. J. Virol..

[28] Reid SP, Valmas C, Martinez O, Sanchez FM, Basler CF (2007). Ebola virus VP24 proteins inhibit the interaction of NPI-1 subfamily karyopherin alpha proteins with activated STAT1. J. Virol..

[29] Reid SP, Leung LW, Hartman AL, Martinez O, Shaw ML, Carbonnelle C, Volchkov VE, Nichol ST, Basler CF (2006). Ebola virus VP24 binds karyopherin alpha1 and blocks STAT1 nuclear accumulation. J. Virol..

[30] Prins KC, Cardenas WB, Basler CF (2009). Ebola virus protein VP35 impairs the function of interferon regulatory factor-activating kinases IKKepsilon and TBK-1. J. Virol..

[31] Cardenas WB, Loo YM, Gale M, Hartman AL, Kimberlin CR, Martinez-Sobrido L, Saphire EO, Basler CF (2006). Ebola virus VP35 protein binds double-stranded RNA and inhibits alpha/beta interferon production induced by RIG-I signaling. J. Virol..

[32] Jin H, Yan Z, Prabhakar BS, Feng Z, Ma Y, Verpooten D, Ganesh B, He B (2010). The VP35 protein of Ebola virus impairs dendritic cell maturation induced by virus and lipopolysaccharide. J. Gen. Virol..

[33] Feng Z, Cerveny M, Yan Z, He B (2007). The VP35 protein of Ebola virus inhibits the antiviral effect mediated by double-stranded RNA-dependent protein kinase PKR. J. Virol..

[34] Hartman AL, Ling L, Nichol ST, Hibberd ML (2008). Whole-genome expression profiling reveals that inhibition of host innate immune response pathways by Ebola virus can be reversed by a single amino acid change in the VP35 protein. J. Virol..

[35] Hartman AL, Bird BH, Towner JS, Antoniadou ZA, Zaki SR, Nichol ST (2008). Inhibition of IRF-3 activation by VP35 is critical for the high level of virulence of ebola virus. J. Virol..

[36] Valmas C, Grosch MN, Schumann M, Olejnik J, Martinez O, Best SM, Krahling V, Basler CF, Muhlberger E (2010). Marburg virus evades interferon responses by a mechanism distinct from ebola virus. PLoS Pathog..

[37] Valmas C, Basler CF (2011). Marburgvirus VP40 antagonizes IFN signaling in a species-specific manner. J. Virol..

[38] Leung LW, Park MS, Martinez O, Valmas C, Lopez CB, Basler CF (2011). Ebolavirus VP35 suppresses IFN production from conventional but not plasmacytoid dendritic cells. Immunol. Cell Biol..

[39] Ramanan PS, R, Leung DW, Basler CF, Amarasinghe GK (2011). Filoviral Immune Evasion Mechanisms. Viruses.

[40] Baize S, Leroy EM, Georges-Courbot MC, Capron M, Lansoud-Soukate J, Debre P, Fisher-Hoch SP, McCormick JB, Georges AJ (1999). Defective humoral responses and extensive intravascular apoptosis are associated with fatal outcome in Ebola virus-infected patients. Nat. Med..

[41] Baize S, Leroy EM, Georges AJ, Georges-Courbot MC, Capron M, Bedjabaga I, Lansoud-Soukate J, Mavoungou E (2002). Inflammatory responses in Ebola virus-infected patients. Clin. Exp. Immunol..

[42] Wauquier N, Becquart P, Padilla C, Baize S, Leroy EM (2010). Human fatal zaire ebola virus infection is associated with an aberrant innate immunity and with massive lymphocyte apoptosis. PLoS Negl. Trop. Dis..

[43] Villinger F, Rollin PE, Brar SS, Chikkala NF, Winter J, Sundstrom JB, Zaki SR, Swanepoel R, Ansari AA, Peters CJ (1999). Markedly elevated levels of interferon (IFN)-gamma, IFN-alpha, interleukin (IL)-2, IL-10, and tumor necrosis factor-alpha associated with fatal Ebola virus infection. J. Infect. Dis..

[44] Couper KN, Blount DG, Riley EM (2008). IL-10: The master regulator of immunity to infection. J. Immunol..

[45] Geisbert TW, Hensley LE, Larsen T, Young HA, Reed DS, Geisbert JB, Scott DP, Kagan E, Jahrling PB, Davis KJ (2003). Pathogenesis of Ebola hemorrhagic fever in cynomolgus macaques: Evidence that dendritic cells are early and sustained targets of infection. Am. J. Pathol..

[46] Hensley LE, Young HA, Jahrling PB, Geisbert TW (2002). Proinflammatory response during Ebola virus infection of primate models: Possible involvement of the tumor necrosis factor receptor superfamily. Immunol. Lett..

[47] Rubins KH, Hensley LE, Wahl-Jensen V, Daddario DiCaprio KM, Young HA, Reed DS, Jahrling PB, Brown PO, Relman DA, Geisbert TW (2007). The temporal program of peripheral blood gene expression in the response of nonhuman primates to Ebola hemorrhagic fever. Genome Biol..

[48] Hutchinson KL, Villinger F, Miranda ME, Ksiazek TG, Peters CJ, Rollin PE (2001). Multiplex analysis of cytokines in the blood of cynomolgus macaques naturally infected with Ebola virus (Reston serotype). J. Med. Virol..

[49] Leroy EM, Baize S, Volchkov VE, Fisher-Hoch SP, Georges-Courbot MC, Lansoud-Soukate J, Capron M, Debre P, McCormick JB, Georges AJ (2000). Human asymptomatic Ebola infection and strong inflammatory response. Lancet.

[50] Leroy EM, Baize S, Debre P, Lansoud-Soukate J, Mavoungou E (2001). Early immune responses accompanying human asymptomatic Ebola infections. Clin. Exp. Immunol..

[51] Ignatyev GM, Agafonov AP, Streltsova MA, Kashentseva EA (1996). Inactivated Marburg virus elicits a nonprotective immune response in Rhesus monkeys. J. Biotechnol..

[52] Ignat'ev GM, Strel'tsova MA, Kashentseva EA, Patrushev NA (1998). Effects of tumor necrosis factor antiserum of the course of Marburg hemorrhagic fever. Vestn. Ross. Akad. Med. Nauk..

[53] Hutchinson KL, Rollin PE (2007). Cytokine and chemokine expression in humans infected with Sudan Ebola virus. J. Infect. Dis..

[54] Sanchez A, Lukwiya M, Bausch D, Mahanty S, Sanchez AJ, Wagoner KD, Rollin PE (2004). Analysis of human peripheral blood samples from fatal and nonfatal cases of Ebola (Sudan) hemorrhagic fever: Cellular responses, virus load, and nitric oxide levels. J. Virol..

[55] Mahanty S, Gupta M, Paragas J, Bray M, Ahmed R, Rollin PE (2003). Protection from lethal infection is determined by innate immune responses in a mouse model of Ebola virus infection. Virology.

[56] Warfield KL, Bosio CM, Welcher BC, Deal EM, Mohamadzadeh M, Schmaljohn A, Aman MJ, Bavari S (2003). Ebola virus-like particles protect from lethal Ebola virus infection. Proc. Natl. Acad. Sci. U. S. A..

[57] (1978). Ebola haemorrhagic fever in Sudan, 1976. Report of a WHO/International Study Team. Bull. World Health Organ..

[58] World Health Organization International Study Team (1978). Ebola haemorrhagic fever in Zaire, 1976. Bull. World Health Organ..

[59] Bradfute SB, Braun DR, Shamblin JD, Geisbert JB, Paragas J, Garrison A, Hensley LE, Geisbert TW (2007). Lymphocyte death in a mouse model of Ebola virus infection. J. Infect. Dis..

[60] Bradfute SB, Warfield KL, Bavari S (2008). Functional CD8+ T cell responses in lethal Ebola virus infection. J. Immunol..

[61] Geisbert TW, Hensley LE, Gibb TR, Steele KE, Jaax NK, Jahrling PB (2000). Apoptosis induced *in vitro* and *in vivo* during infection by Ebola and Marburg viruses. Lab. Invest..

[62] Reed DS, Hensley LE, Geisbert JB, Jahrling PB, Geisbert TW (2004). Depletion of peripheral blood T lymphocytes and NK cells during the course of ebola hemorrhagic Fever in cynomolgus macaques. Viral. Immunol..

[63] Baize S, Leroy EM, Mavoungou E, Fisher-Hoch SP (2000). Apoptosis in fatal Ebola infection. Does the virus toll the bell for immune system?. Apoptosis.

[64] Gibb TR, Bray M, Geisbert TW, Steele KE, Kell WM, Davis KJ, Jaax NK (2001). Pathogenesis of experimental Ebola Zaire virus infection in BALB/c mice. J. Comp. Pathol..

[65] Geisbert TW, Young HA, Jahrling PB, Davis KJ, Larsen T, Kagan E, Hensley LE (2003). Pathogenesis of Ebola hemorrhagic fever in primate models: Evidence that hemorrhage is not a direct effect of virus-induced cytolysis of endothelial cells. Am. J. Pathol..

[66] Ksiazek TG, West CP, Rollin PE, Jahrling PB, Peters CJ (1999). ELISA for the detection of antibodies to Ebola viruses. J. Infect. Dis..

[67] Onyango CO, Opoka ML, Ksiazek TG, Formenty P, Ahmed A, Tukei PM, Sang RC, Ofula VO, Konongoi SL, Coldren RL (2007). Laboratory diagnosis of Ebola hemorrhagic fever during an outbreak in Yambio, Sudan, 2004. J. Infect. Dis..

[68] Towner JS, Rollin PE, Bausch DG, Sanchez A, Crary SM, Vincent M, Lee WF, Spiropoulou CF, Ksiazek TG, Lukwiya M (2004). Rapid diagnosis of Ebola hemorrhagic fever by reverse transcription-PCR in an outbreak setting and assessment of patient viral load as a predictor of outcome. J. Virol..

[69] Bradfute SB, Swanson PE, Smith MA, Watanabe E, McDunn JE, Hotchkiss RS, Bavari S (2010). Mechanisms and consequences of ebolavirus-induced lymphocyte apoptosis. J. Immunol..

[70] Jiang J, Lau LL, Shen H (2003). Selective depletion of nonspecific T cells during the early stage of immune responses to infection. J. Immunol..

[71] McNally JM, Zarozinski CC, Lin MY, Brehm MA, Chen HD, Welsh RM (2001). Attrition of bystander CD8 T cells during virus-induced T-cell and interferon responses. J. Virol..

[72] Warfield KL, Swenson DL, Demmin G, Bavari S (2005). Filovirus-like particles as vaccines and discovery tools. Expert. Rev. Vaccines.

[73] Warfield KL, Swenson DL, Olinger GG, Kalina WV, Aman MJ, Bavari S (2007). Ebola virus-like particle-based vaccine protects nonhuman primates against lethal Ebola virus challenge. J. Infect. Dis..

[74] Jones SM, Feldmann H, Stroher U, Geisbert JB, Fernando L, Grolla A, Klenk HD, Sullivan NJ, Volchkov VE, Fritz EA (2005). Live attenuated recombinant vaccine protects nonhuman primates against Ebola and Marburg viruses. Nat. Med..

[75] Hevey M, Negley D, Pushko P, Smith J, Schmaljohn A (1998). Marburg virus vaccines based upon alphavirus replicons protect guinea pigs and nonhuman primates. Virology.

[76] Bukreyev A, Rollin PE, Tate MK, Yang L, Zaki SR, Shieh WJ, Murphy BR, Collins PL, Sanchez A (2007). Successful topical respiratory tract immunization of primates against Ebola virus. J. Virol..

[77] Sullivan NJ, Geisbert TW, Geisbert JB, Xu L, Yang ZY, Roederer M, Koup RA, Jahrling PB, Nabel GJ (2003). Accelerated vaccination for Ebola virus haemorrhagic fever in non-human primates. Nature.

[78] Sullivan NJ, Sanchez A, Rollin PE, Yang ZY, Nabel GJ (2000). Development of a preventive vaccine for Ebola virus infection in primates. Nature.

[79] Hensley LE, Mulangu S, Asiedu C, Johnson J, Honko AN, Stanley D, Fabozzi G, Nichol ST, Ksiazek TG, Rollin PE (2010). Demonstration of cross-protective vaccine immunity against an emerging pathogenic Ebolavirus Species. PLoS Pathog..

[80] Sullivan NJ, Martin JE, Graham BS, Nabel GJ (2009). Correlates of protective immunity for Ebola vaccines: Implications for regulatory approval by the animal rule. Nat. Rev. Microbiol..

[81] Daddario-DiCaprio KM, Geisbert TW, Geisbert JB, Stroher U, Hensley LE, Grolla A, Fritz EA, Feldmann F, Feldmann H, Jones SM (2006). Cross-protection against Marburg virus strains by using a live, attenuated recombinant vaccine. J. Virol..

[82] Daddario-DiCaprio KM, Geisbert TW, Stroher U, Geisbert JB, Grolla A, Fritz EA, Fernando L, Kagan E, Jahrling PB, Hensley LE (2006). Postexposure protection against Marburg haemorrhagic fever with recombinant vesicular stomatitis virus vectors in non-human primates: An efficacy assessment. Lancet.

[83] Feldmann H, Jones SM, Daddario-DiCaprio KM, Geisbert JB, Stroher U, Grolla A, Bray M, Fritz EA, Fernando L, Feldmann F (2007). Effective post-exposure treatment of Ebola infection. PLoS Pathog..

[84] Geisbert TW, Geisbert JB, Leung A, Daddario-DiCaprio KM, Hensley LE, Grolla A, Feldmann H (2009). Single-injection vaccine protects nonhuman primates against infection with marburg virus and three species of ebola virus. J. Virol..

[85] Geisbert TW, Hensley LE, Geisbert JB, Leung A, Johnson JC, Grolla A, Feldmann H (2010). Postexposure treatment of Marburg virus infection. Emerg. Infect. Dis..

[86] Jones S, Geisbert T, Stroher U, Geisbert J, Bray M, Sullivan N, Jahrling P, Feldmann H Replicating vectors for vaccine development.

[87] Warfield KL, Olinger G, Deal EM, Swenson DL, Bailey M, Negley DL, Hart MK, Bavari S (2005). Induction of humoral and CD8+ T cell responses are required for protection against lethal Ebola virus infection. J. Immunol..

[88] Mupapa K, Massamba M, Kibadi K, Kuvula K, Bwaka A, Kipasa M, Colebunders R, Muyembe-Tamfum JJ (1999). Treatment of Ebola hemorrhagic fever with blood transfusions from convalescent patients. International Scientific and Technical Committee. J. Infect. Dis..

[89] Wilson JA, Hevey M, Bakken R, Guest S, Bray M, Schmaljohn AL, Hart MK (2000). Epitopes involved in antibody-mediated protection from Ebola virus. Science.

[90] Wilson JA, Bray M, Bakken R, Hart MK (2001). Vaccine potential of Ebola virus VP24, VP30, VP35, and VP40 proteins. Virology.

[91] Jones SM, Stroher U, Fernando L, Qiu X, Alimonti J, Melito P, Bray M, Klenk HD, Feldmann H (2007). Assessment of a vesicular stomatitis virus-based vaccine by use of the mouse model of Ebola virus hemorrhagic fever. J. Infect. Dis..

[92] Takada A, Ebihara H, Jones S, Feldmann H, Kawaoka Y (2007). Protective efficacy of neutralizing antibodies against Ebola virus infection. Vaccine.

[93] Parren PW, Geisbert TW, Maruyama T, Jahrling PB, Burton DR (2002). Pre- and postexposure prophylaxis of Ebola virus infection in an animal model by passive transfer of a neutralizing human antibody. J. Virol..

[94] Borisevich IV, Potryvaeva NV, Mel'nikov SA, Evseev AA, Krasnianskii VP, Maksimov VA (2008). Design of equine serum-based Marburg virus immunoglobulin. Vopr. Virusol..

[95] Hevey M, Negley D, Schmaljohn A (2003). Characterization of monoclonal antibodies to Marburg virus (strain Musoke) glycoprotein and identification of two protective epitopes. Virology.

[96] Razumov IA, Belanov EF, Bormotov NI, Kazachinskaia EI (2001). Detection of antiviral activity of monoclonal antibodies, specific to Marburg virus proteins. Vopr. Virusol..

[97] Kudoyarova-Zubavichene NM, Sergeyev NN, Chepurnov AA, Netesov SV (1999). Preparation and use of hyperimmune serum for prophylaxis and therapy of Ebola virus infections. J. Infect. Dis..

[98] Gupta M, Mahanty S, Bray M, Ahmed R, Rollin PE (2001). Passive transfer of antibodies protects immunocompetent and imunodeficient mice against lethal Ebola virus infection without complete inhibition of viral replication. J. Virol..

[99] Borisevich IV, Mikhailov VV, Krasnianskii VP, Gradoboev VN, Lebedinskaia EV, Potryvaeva NV, Timan'kova GD (1995). Development and study of the properties of immunoglobulin against Ebola fever. Vopr. Virusol..

[100] Markin VA, Mikhailov VV, Krasnianskii VP, Borisevich IV, Firsova IV (1997). Developing principles for emergency prevention and treatment of Ebola fever. Vopr. Virusol..

[101] Jahrling PB, Geisbert JB, Swearengen JR, Larsen T, Geisbert TW (2007). Ebola hemorrhagic fever: Evaluation of passive immunotherapy in nonhuman primates. J. Infect. Dis..

[102] Oswald WB, Geisbert TW, Davis KJ, Geisbert JB, Sullivan NJ, Jahrling PB, Parren PW, Burton DR (2007). Neutralizing antibody fails to impact the course of Ebola virus infection in monkeys. PLoS Pathog..

[103] Gupta M, Greer P, Mahanty S, Shieh WJ, Zaki SR, Ahmed R, Rollin PE (2005). CD8-mediated protection against Ebola virus infection is perforin dependent. J. Immunol..

[104] Gupta M, Mahanty S, Greer P, Towner JS, Shieh WJ, Zaki SR, Ahmed R, Rollin PE (2004). Persistent infection with ebola virus under conditions of partial immunity. J. Virol..

[105] Olinger GG, Bailey MA, Dye JM, Bakken R, Kuehne A, Kondig J, Wilson J, Hogan RJ, Hart MK (2005). Protective cytotoxic T-cell responses induced by venezuelan equine encephalitis virus replicons expressing Ebola virus proteins. J. Virol..

[106] Kalina WV, Warfield KL, Olinger GG, Bavari S (2009). Discovery of common marburgvirus protective epitopes in a BALB/c mouse model. Virol. J..

[107] Sullivan NJ, Geisbert TW, Geisbert JB, Shedlock DJ, Xu L, Lamoreaux L, Custers JH, Popernack PM, Yang ZY, Pau MG (2006). Immune protection of nonhuman primates against Ebola virus with single low-dose adenovirus vectors encoding modified GPs. PLoS Med..

[108] Geisbert TW, Bailey M, Geisbert JB, Asiedu C, Roederer M, Grazia-Pau M, Custers J, Jahrling P, Goudsmit J, Koup R (2010). Vector choice determines immunogenicity and potency of genetic vaccines against Angola Marburg virus in nonhuman primates. J. Virol..

[109] Warfield KL, Perkins JG, Swenson DL, Deal EM, Bosio CM, Aman MJ, Yokoyama WM, Young HA, Bavari S (2004). Role of natural killer cells in innate protection against lethal ebola virus infection. J. Exp. Med..

[110] Fuller CL, Ruthel G, Warfield KL, Swenson DL, Bosio CM, Aman MJ, Bavari S (2007). NKp30-dependent cytolysis of filovirus-infected human dendritic cells. Cell. Microbiol..

[111] Wauquier N, Padilla C, Becquart P, Leroy E, Vieillard V (2010). Association of KIR2DS1 and KIR2DS3 with fatal outcome in Ebola virus infection. Immunogenetics.

[112] Sanchez A, Wagoner KE, Rollin PE (2007). Sequence-based human leukocyte antigen-B typing of patients infected with Ebola virus in Uganda in 2000: Identification of alleles associated with fatal and nonfatal disease outcomes. J. Infect. Dis..

[113] Gibb TR, Norwood DA, Woollen N, Henchal EA (2002). Viral replication and host gene expression in alveolar macrophages infected with Ebola virus (Zaire strain). Clin. Diagn. Lab. Immunol..

[114] Stroher U, West E, Bugany H, Klenk HD, Schnittler HJ, Feldmann H (2001). Infection and activation of monocytes by Marburg and Ebola viruses. J. Virol..

[115] Wyers M, Formenty P, Cherel Y, Guigand L, Fernandez B, Boesch C, Le Guenno B (1999). Histopathological and immunohistochemical studies of lesions associated with Ebola virus in a naturally infected chimpanzee. J. Infect. Dis..

[116] Ryabchikova EI, Kolesnikova LV, Luchko SV (1999). An analysis of features of pathogenesis in two animal models of Ebola virus infection. J. Infect. Dis..

[117] Geisbert TW, Jahrling PB, Hanes MA, Zack PM (1992). Association of Ebola-related Reston virus particles and antigen with tissue lesions of monkeys imported to the United States. J. Comp. Pathol..

[118] Davis KJ, Anderson AO, Geisbert TW, Steele KE, Geisbert JB, Vogel P, Connolly BM, Huggins JW, Jahrling PB, Jaax NK (1997). Pathology of experimental Ebola virus infection in African green monkeys. Involvement of fibroblastic reticular cells. Arch. Pathol. Lab. Med..

[119] Geisbert TW, Jaax NK (1998). Marburg hemorrhagic fever: Report of a case studied by immunohistochemistry and electron microscopy. Ultrastruct. Pathol..

[120] Bosio CM, Aman MJ, Grogan C, Hogan R, Ruthel G, Negley D, Mohamadzadeh M, Bavari S, Schmaljohn A (2003). Ebola and Marburg viruses replicate in monocyte-derived dendritic cells without inducing the production of cytokines and full maturation. J. Infect. Dis..

[121] Mahanty S, Hutchinson K, Agarwal S, McRae M, Rollin PE, Pulendran B (2003). Cutting edge: Impairment of dendritic cells and adaptive immunity by Ebola and Lassa viruses. J. Immunol..

[122] Leung LWM, O, Reynard O, Volchkov VE, Basler C (2011). Ebola virus failure to stimulate plasmacytoid dendritic cell interferon responses correlates with impaired cellular entry. J. Infect. Dis..

[123] Bray M, Geisbert TW (2005). Ebola virus: The role of macrophages and dendritic cells in the pathogenesis of Ebola hemorrhagic fever. Int. J. Biochem. Cell Biol..

[124] Geisbert TW, Hensley LE, Kagan E, Yu EZ, Geisbert JB, Daddario-DiCaprio K, Fritz EA, Jahrling PB, McClintock K, Phelps JR (2006). Postexposure protection of guinea pigs against a lethal ebola virus challenge is conferred by RNA interference. J. Infect. Dis..

[125] Warren TK, Warfield KL, Wells J, Enterlein S, Smith M, Ruthel G, Yunus AS, Kinch MS, Goldblatt M, Aman MJ (2010). Antiviral activity of a small-molecule inhibitor of filovirus infection. Antimicrob. Agents Chemother..

